# Symptoms of Intestinal Obstruction

**Published:** 1878-04

**Authors:** 


Symptoms of Intestinal Obstruction.—It is erroneous, observes Hensgen (*Deutsche Medicin. Wochen,* December, 1877,) to assume that obstruction is always accompanied by fæcal vomiting. This is impossible in occlusion of the upper portions of the small intestine, nor has it been confirmed in all cases of closure of the large bowel. In illustration of the latter fact, Hensgen cites a remarkable case of ileus, the most striking feature of which was the long duration of complete closure of the descending colon, namely, forty-four days, during which time nothing passed from the bowel, and also an entire absence of any escape of the intestinal contents upwards, either fæcal, or gas having a fæcal odour. The absence of fæcal vomiting must be regarded as due to the action of the ilio-cæcal valve, which also sufficiently accounts for the non-distension of the small intestine observed in this case.—*Med. Press and Circular.*

